# Exploring Vacuum Compression Molding as a Preparation Method for Flexible-Dose Pediatric Orodispersible Films

**DOI:** 10.3390/ph17070934

**Published:** 2024-07-12

**Authors:** Dana Hales, Cătălina Bogdan, Lucia Ruxandra Tefas, Andreea Cornilă, Maria-Andreea Chiver, Ioan Tomuță, Tibor Casian, Rareș Iovanov, Gábor Katona, Rita Ambrus, Sonia Iurian

**Affiliations:** 1 Department of Pharmaceutical Technology and Biopharmacy, Iuliu Hatieganu University of Medicine and Pharmacy, 41 Victor Babeș St, 400002 Cluj-Napoca, Romania; dudas.dana@umfcluj.ro (D.H.); tefas.lucia@umfcluj.ro (L.R.T.); ioana.an.cornila@elearn.umfcluj.ro (A.C.); maria.andr.chiver@elearn.umfcluj.ro (M.-A.C.); tomutaioan@umfcluj.ro (I.T.); casian.tibor@umfcluj.ro (T.C.); riovanov@umfcluj.ro (R.I.); sonia.iurian@umfcluj.ro (S.I.); 2 Department of Dermopharmacy and Cosmetology, Iuliu Hatieganu University of Medicine and Pharmacy, 12 Ion Creangă St, 400002 Cluj-Napoca, Romania; 3 Institute of Pharmaceutical Technology and Regulatory Affairs, University of Szeged, Eotvos u. 6, 6720 Szeged, Hungary; katona.gabor@szte.hu (G.K.); ambrus.rita@szte.hu (R.A.)

**Keywords:** vacuum compression molding, orodispersible films, diclofenac sodium, pediatric formulations

## Abstract

In recent years, solid dosage forms have gained interest in pediatric therapy because they can provide valuable benefits in terms of dose accuracy and stability. Particularly for orodispersible films (ODFs), the literature evidences increased acceptability and dose flexibility. Among the various available technologies for obtaining ODFs, such as solvent casting, hot-melt extrusion, and ink printing technologies, the solvent-free preparation methods exhibit significant advantages. This study investigated Vacuum Compression Molding (VCM) as a solvent-free manufacturing method for the preparation of flexible-dose pediatric orodispersible films. The experimental approach focused on selecting the appropriate plasticizer and ratios of the active pharmaceutical ingredient, diclofenac sodium, followed by the study of their impacts on the mechanical properties, disintegration time, and drug release profile of the ODFs. Additional investigations were performed to obtain insights regarding the solid-state properties. The ODFs obtained by VCM displayed adequate quality in terms of their critical characteristics. Therefore, this proof-of-concept study shows how VCM could be utilized as a standalone method for the production of small-scale ODFs, enabling the customization of doses to meet the individual needs of pediatric patients.

## 1. Introduction

The recent initiatives of the regulatory authorities provide a dynamic landscape for pediatric medicines, encouraging the development of high-quality and innovative formulations at affordable costs. The importance of research on novel delivery systems and formulation approaches for pediatric medicines is therefore emphasized [1,2]. Yet, these objectives pose financial challenges for the pharmaceutical industry due to the necessity to develop formulations with distinct characteristics for specific age groups, such as neonates, infants, children, or adolescents [3]. Moreover, the scarcity of data on patient preferences and acceptability for emerging dosage forms within the highly diverse pediatric population poses the challenge of choosing between different formulation approaches. Therefore, given the limited availability of tailored pediatric medicines, the adjustment of products manufactured by the pharmaceutical industry for the personalization of medication in the case of specific groups of patients, such as children, as well as extemporaneous compounding, have become routine practices [4]. Unfortunately, the intervention of the pharmacist in the pharmaceutical forms prepared for adults in order to transform them into pediatric pharmaceutical forms with specific API doses, or with certain shapes or sizes, may lead to a high risk of dosing errors. Nevertheless, extemporaneous compounding is regarded as a convenient option to obtain acceptable dosage forms customized for a limited number of pediatric patients or during drug shortages. Compounded pediatric medicines are regularly available as liquid dosage forms or as powders intended for reconstitution. However, these dosage forms often have inherent limitations concerning their stability and taste.

Recently, the trend has been shifting towards replacing liquid dosage forms with solid ones due to several benefits, such as more accurate dosing, better stability, lower costs, and increasing evidence supporting better acceptability [5]. Through the collaborative efforts of the authorities and different research groups, significant progress has been made in developing age-appropriate solid formulations. In recent years, numerous innovative technologies and delivery systems with additional benefits have been proposed, but significant gaps still exist. Among them, orodispersible films (ODFs) are versatile dosage forms offering significant benefits in terms of dosing flexibility and acceptability [6]. They can be administered without water, and despite being a relatively new technology platform, several commercial products are available.

ODF manufacturing methods include solvent casting, hot-melt extrusion (HME), electrospinning, and ink printing technologies [3]. HME is a promising solvent-free technology, generally employed for APIs that display water sensitivity [7]. Moreover, an important application of HME is the preparation of amorphous solid dispersions (ASDs) from API–polymer mixtures. Their use is typically associated with improved solubility and the bioavailability of poorly soluble APIs, resulting in a faster onset of action [8]. With a better understanding of HME and amorphous systems, new technologies are continuously being explored to match individual patient needs, such as 3D printing and ODF extrusion. However, the implementation of HME involves several complex stages, and successful development requires experimental work, trained personnel [9], and significant quantities of raw materials [10,11]. Moreover, these new technologies raise concerns about the costs related to their manufacturing, as well as regulatory challenges [12]. Regarding the regulatory aspects, while some believe that a clear implementation of good manufacturing practice (GMP) and the International Organization for Standardization (ISO) is needed in order to obtain products of high and consistent quality [12], others consider that the regulatory and quality assurance for these products is comparable to that of standard magistral products [13]. Regarding the costs, it seems that personalized medicines have the potential to be cost-effective, due to the possibility of manufacturing small and versatile batches, even if they may vary widely depending on the equipment used and the country [14,15].

First applied as a sample preparation technique, Vacuum Compression Molding (VCM) is a fusion-based method used to obtain compact, HME-like samples from powders. Applying vacuum and heat, the API is melted or solubilized into a polymer, resulting in an ASD. Samples obtained via VCM are transparent discs or bars containing the amorphous form of an API incorporated in the polymeric matrix [3]. VCM was developed as a preformulation tool for quick and cost-effective ASD development, enabling the easy preparation of samples for further stability and performance testing [10]. Lately, it has been applied as a lossless processing technique to obtain small-scale formulations [10]. So far, the technique has been employed in different applications, such as the preparation of polymeric microneedles for transdermal drug delivery [16], the study of the efavirenz solubility limits in different matrix polymers [17], as a protein-stabilizing method for potential HME processing [18], or for the preparation of subcutaneous implants [19].

In pediatric therapy, there is a constant need for new preparation methods with simple working principles that deliver customized pharmaceutical products; thus, this proof-of-principle study explores the use of VCM in ODF production. The tested hypothesis is that VCM could act as a method of preparing small-scale pediatric products that is accessible to community or hospital pharmacies. The data currently available show that there is increasing interest in the introduction of personalized medicines in therapy, with one of the recurring applications being the use of this technique in clinical studies [20]. A 2021 study investigated how 3D printing technologies can be implemented in the European pharmaceutical system (i.e., the Netherlands). Of the five scenarios investigated to assess issues that could affect the implementation, industry and the patient’s homes were associated with the most challenges, while hospital pharmacies and compounding facilities were associated with the fewest [21]. Although the prospects are encouraging for manufacturing personalized medicines in hospital pharmacies and compounding facilities, there are still no international policies or guidelines to provide a framework in this respect [22]. For now, the personalized products are manufactured in accordance with the standard compounding regulations and are subject to quality, safety, and stability control to prove that they are suitable for patient administration [20].

In this study, the experimental approach focused on the plasticizer selection and API (diclofenac sodium) loading with varying ratios to yield the appropriate mechanical characteristics, disintegration, and drug release profile for the ODFs. Further investigations were performed to assess the solid-state properties. To the best of our knowledge, so far, there are no reports on VCM employed for the production of small-scale ODFs.

## 2. Results and Discussion

In this study, VCM technology was used to prepare ODFs for pediatric use. The careful selection of ingredients is a prerequisite to ensuring the product performance and acceptability.

Maltodextrin (MD) was chosen for its well-known functionality as an orodispersible-film-forming agent, which is true, especially for the sorts with dextrose equivalents between 6 and 12 [23,24]. Further, the plasticizers were chosen based on information gathered from the literature, such as the non-miscibility of MD with PEG 400 and citric acid esters [25]. Cilurzo et al. showed that MD was compatible with glycerol and propylene glycol, which acted as efficient plasticizers [25]. However, propylene glycol imparted an unpleasant taste to the films, according to data collected from volunteers [26]. Regarding glycerol, during the screening stage of the present study, there were attempts to introduce this plasticizing agent into the formulations, but it was not possible to obtain appropriate films. Other frequently used plasticizers for improving the ductility of maltodextrin ODFs are sorbitol and xylitol; therefore, they were included in the study [26]. Given the bitter taste of diclofenac sodium, the addition of plasticizers with sweetener properties increased the acceptability of the formulation [27]. Mannitol is not a first-choice plasticizer for ODF preparation, but due to the similar structure to that of sorbitol and xylitol, it was also considered a worthy option to investigate, considering that mannitol may influence the mechanical properties of the ODFs [6].

Based on the data collected in the preliminary stage, loaded ODFs were prepared with increasing diclofenac sodium concentrations, maltodextrin as a film-forming agent, xylitol as a plasticizer, and croscarmellose as a superdisintegrant.

### 2.1. Appearance and Size

The vacuum-molding process resulted in round-shaped, completely translucent films, slightly yellow in color, at increasing diclofenac sodium doses (as shown in Table 1 for formulations N5–N8), with a diameter of 20 mm. When compared to commercial ODFs, they are slightly smaller in size, thicker, and more transparent.

### 2.2. Mechanical Characterization

Puncture tests are the most frequently cited in the literature for evaluating the mechanical properties of orodispersible films. These tests rely on fixing the sample between two plates and measuring the load needed for a probe to puncture the sample through a central orifice [28]. As mentioned by other authors [29], the gap is usually similar in size or larger compared to those of commercial film samples. As the ODFs prepared in this study were smaller than most commercial ODFs, a customized accessory with a smaller gap diameter was used. Moreover, considering the means of unpacking and manipulating the ODFs prior to administration, their mechanical properties were also assessed through tensile tests [30]. Although the two tests can lead to similar mechanical parameters, many authors consider that they can complement each other in explaining the mechanical behavior of ODFs [31]. Table 2 presents the ODF parameters obtained from the mechanical tests.

Formulations N1–N3 contained MD and one of three plasticizers: sorbitol, xylitol, and mannitol, respectively. Although it would have been interesting to highlight the differences in the mechanical features between the samples containing plasticizers and those made of MD alone, the preparation and mechanical testing of the MD films were not feasible, as the MD did not melt at the VCM working temperature employed in this study.

**Figure 1:**
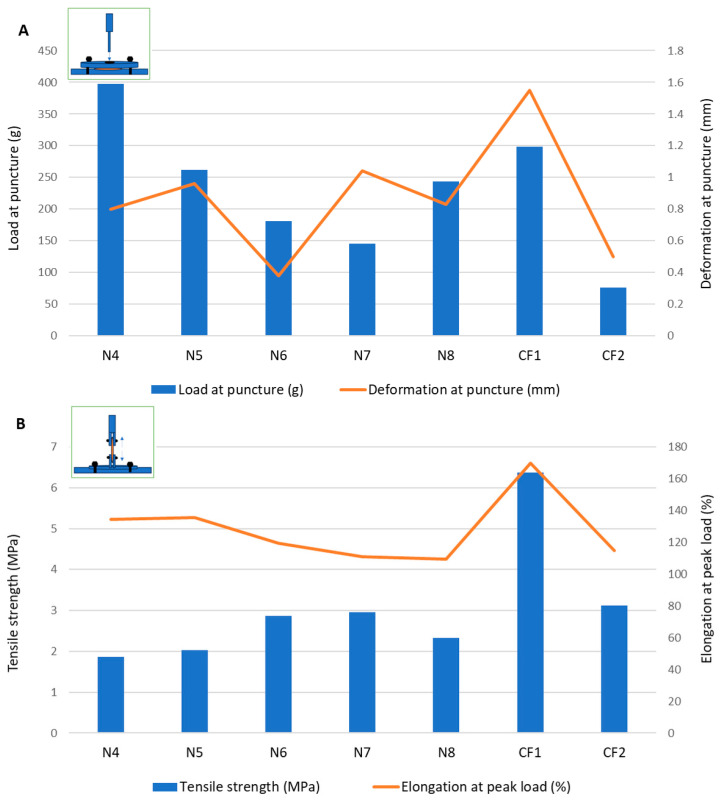
Mechanical characterization of films through (**A**) puncture test and (**B**) tensile test.

The puncture loads obtained for the samples containing MD and the plasticizer mixtures without the disintegrant (N1–N3) ranged between 94.5 ± 9.00 g and 300.30 ± 109.80 g, which showed a promising overlap with the load values for the commercial ODF products (Figure 1). Sorbitol showed the best performance in terms of both the puncture load and flexibility, indicated by the highest puncture deformation (0.88 ± 0.37 mm). Mannitol led to the most rigid structure in terms of the intermediate load at puncture, but it allowed for a very low deformation of only 0.09 ± 0.04 mm. In the puncture test, the N2 sample containing xylitol showed the lowest puncture load and lowest fracturability (load at the first fracture), but it allowed for a higher deformation compared to that of the mannitol. Looking at the tensile test results for formulations N1–N3, using xylitol as a plasticizer led to the highest tensile strength values, meaning that the films containing xylitol needed the highest forces to be deformed by tension. This led to the selection of xylitol as a plasticizer in the further formulations (from N4 to N8).

In formulation N4, the croscarmellose, added for its superdisintegrant properties, also positively impacted the mechanical features of the films. During the puncture test, the puncture load increased significantly compared to formulation N2 without croscarmellose, along with the deformation at puncture and fracturability. Therefore, the croscarmellose improved the flexibility of the films, as well as their resistance to fractures. The breaking factor, which is regarded as the most appropriate parameter to consider when comparing samples with different thicknesses [29], had the highest value for N4.

The results obtained for the VCM ODFs indicate different responses according to the forces applied. For example, the ODF drug loading decreased the load needed to puncture the samples, regardless of the API dose, while it determined an increase in the tensile strength when the same samples were extended.

**Figure 2:**
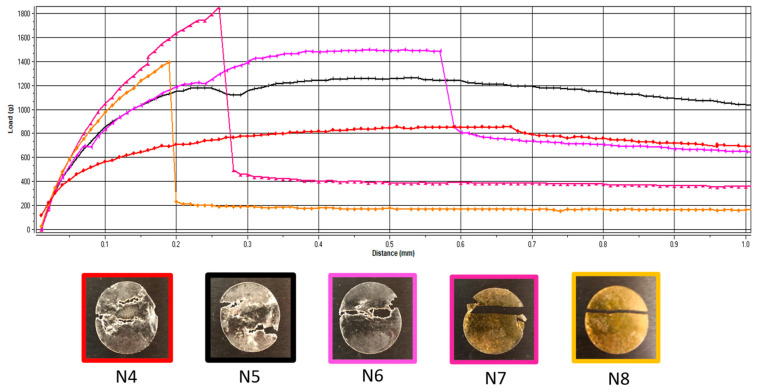
Representative load-versus-distance profiles of VCM ODFs and associated images of the samples at the end of the tensile test. VCM ODF formulations are represented in different colors: N4: red; N5: black; N6: pink; N7: magenta; N8: yellow.

Additionally, the deformation obtained in the puncture test had a random variation with the lowest value corresponding to the lowest flexibility recorded for N6 (with 5 mg of API per unit) and the maximum deformation for N7 (with 10 mg of API per unit) (Figure 1). In contrast, for the tensile test, the deformation values were not influenced by lower API doses, but when the dose increased, the deformation capacity of the films decreased along with the dose. Figure 2 depicts the appearance of the ODFs submitted to the tensile tests after a constant deformation of 16.6%. The placebo sample (N4) and the low-dose diclofenac sodium formulations (N5 and N6) exhibited neck formation and elastic breaks, while brittle fractures appeared at high drug loading for N7 and N8, which also displayed higher Young’s moduli. Therefore, adding the API resulted in less ductile films, which allowed for lower deformation when they were subjected to tensile stress. Our findings are in agreement with the results obtained by other researchers for maltodextrin-based, solvent-cast ODFs [23].

Since there are no official limits for the mechanical parameters that describe ODFs, two authorized commercial products obtained by solvent casting were evaluated under the same conditions. The load results for the VCM ODFs were largely in the same range or higher than those for the commercial products, at all the drug loadings and regardless of the applied test. In contrast, for the deformation capacity, values lower than those of any commercial product were obtained for formulations with high drug loadings (between 10 mg and 50 mg per unit) during the tensile testing. Other researchers have attempted to establish acceptance criteria for the puncture strengths of different types of orodispersible films. The authors of [32] found values over 0.08 N/mm^2^ acceptable for 3D printed films, while the authors of [33] mentioned values over 0.06 N/mm^2^ for solvent-cast films [32,33]. This indicates that VCM ODFs are well within the acceptable limits. Moreover, the authors of [25] reported values in the same ranges for their HME films containing MD and piroxicam as an API [25].

### 2.3. Contact Angle Measurements

**Figure 3:**
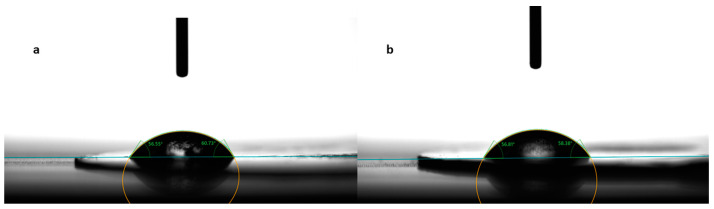
Contact angle measurement for formulations N2 (**a**) and N5 (**b**).

Contact angle evaluation was used as a tool to predict the wettability and further disintegration of the ODFs [34]. The contact angle values for the eight formulations are noted in Table 3, expressed as the mean ± S.D. of three measurements. Figure 3 shows photographs taken during the measurements for the formulations N2, without diclofenac sodium, and N5, with 2.5 mg diclofenac sodium. Among the films containing only MD and a plasticizer, the smallest contact angle was reported for N3 (42.10 ± 4.72°), containing mannitol as the plasticizer, followed by N2 (47.39 ± 9.77°), containing xylitol as the plasticizer, and N1 (52.14 ± 2.38°), containing sorbitol. The results are consistent with the water solubilities of the three plasticizers, namely, 1:0.5 for sorbitol, 1:1.6 for xylitol, and 1:5.5 for mannitol [35]. Noticeably, the increase in the solubility of the plasticizer led to smaller values for the contact angle.

Regarding formulation N4, even though croscarmellose is insoluble in water, the addition of this disintegrant did not change the hydrophilicity of the films, and the contact angles remained almost unchanged (47.44 ± 6.08° for N4 and 47.39 ± 9.77° for N2).

The drug-loaded films showed varying values for the contact angle, as indicated in Table 3. Compared to formulation N4, which did not contain any API, the addition of diclofenac sodium imparted slight hydrophobicity to the films, evidenced by the larger values for the contact angles for formulations N5–N8. However, the contact angle decreased with the increase in the diclofenac sodium concentration. Even though diclofenac sodium is a sparingly hydrosoluble salt, at lower concentrations, it led to the highest contact angle (64.83 ± 7.98° for N5). Interestingly, as its concentration increased, it imprinted a hydrophilic character on the obtained films and, implicitly, a decrease in the contact angle (from 64.83 ± 7.98° for N5 to 47.90 ± 5.49° for N8).

The Ph. Eur.’s provisions regarding the wettability of solids are that “wettable solids should show a low contact angle and non-wettable solids show a contact angle of 90° or more” [36]. Therefore, the average contact angle of ODFs ranging between 42.10 ± 4.72° and 64.83 ± 7.98° indicates some degree of hydrophilicity [37].

### 2.4. Disintegration Behavior

The disintegration times of the ODFs, according to the pharmacopeial and texture analysis methods, are illustrated in Table 3. Regarding the disintegration times obtained under pharmacopeial conditions, the values ranged from 60.00 ± 0 s to 101.67 ± 20.21 s. Ph. Eur. does not have specific requirements for orodispersible films; the only information provided is that they should “disperse rapidly” after being placed on the tongue [38]. Therefore, the results were compared with the Ph. Eur. requirements for orodispersible tablets, namely, disintegration “within 3 min, using water as the liquid medium” [39], and the results show that, without a doubt, all the formulations complied with these exigencies. The films containing only MD and a plasticizer (N1–N3) had the shortest disintegration times of about 60–70 s. The addition of the disintegrant, croscarmellose, did not increase the disintegration time significantly, but the subsequent addition of diclofenac sodium led to an increase of approximately 40 s for the formulations containing low drug concentrations (N5 and N6). However, further increasing the diclofenac sodium concentration (N7 and N8) resulted in shortening the disintegration times down to values similar to those of drug-free films.

When the conditions in the oral cavity were simulated in the texture analysis method, as expected, the disintegration times were shorter than those measured according to the pharmacopeial method. Similar results were obtained by the authors of [30], who reported that less was time needed when using the non-pharmacopeial method by better mimicking the disintegration process in the human mouth (i.e., using simulated salivary fluid in a reduced volume) [30]. These findings might suggest that the pressure applied to the product and the characteristics of the medium used (pH, composition) are more important than its volume. In our study, the disintegration times fell between 41 ± 14.25 s and 65.33 ± 10.27 s. Although no significant differences were obtained between the formulations, a decreasing trend in the disintegration times was observed with the increase in the concentration of diclofenac sodium. This can be explained by the increased hydrophilicity and better wetting capacity, also reflected by the contact angle measurements. Thus, the results obtained using texture analysis are in accordance with the previously mentioned results obtained using the pharmacopeial method. The disintegration times of the VCM ODFs are well within the pharmacopeial limit of 180 s and seem robust concerning the API dose changes, which is beneficial for the preparation of small-scale personalized products.

The authors of [25] obtained similar results, with a slower disintegration of approximately 1 min for the HME films (containing piroxicam and MD) compared to the solvent-cast films with a disintegration of a few seconds [25]. The authors of [24] also obtained an average disintegration time of about 1 min for diclofenac-loaded maltodextrin orodispersible films [40].

### 2.5. In Vitro Dissolution Studies

**Figure 4:**
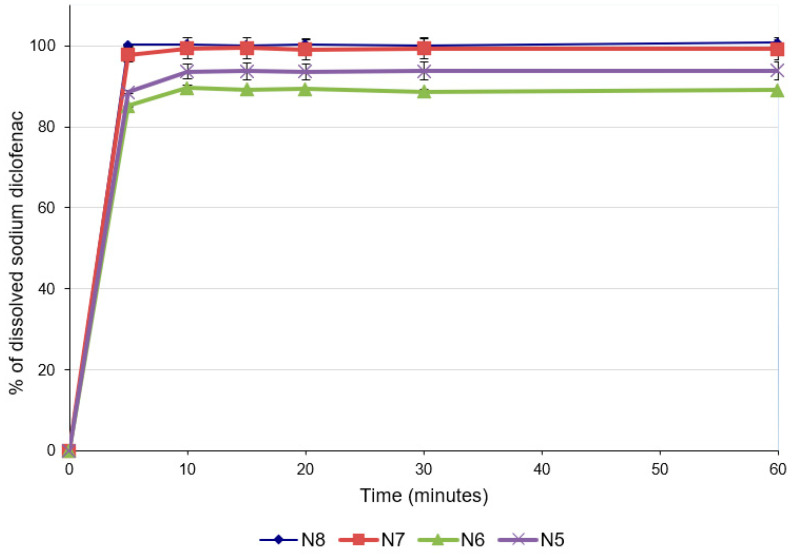
Dissolution profiles of ODFs with diclofenac sodium.

For predicting the in vivo dissolution behavior of the ODFs, an in vitro test was performed using the pharmacopeial method for immediate-release tablets, since there are no official compendial methodologies for ODF dissolution studies. All formulations released more than 85% of the API within 5 min (Figure 4), which accounts for very rapidly dissolving products [41,42]. The concentration of diclofenac sodium released was influenced by the concentration of the drug in the formulation. Thus, the formulations with lower concentrations of diclofenac sodium released lower concentrations of the drug after 5 min (88.56 ± 0.30% for N5 and 85.11 ± 0.01% for N6), while for the last two formulations with higher diclofenac sodium contents, higher percentages of drug release were noticed after 5 min (97.83 ± 1.92% for N7 and 100.22 ± 0.15% for N8). These results are also valid for the total amount of diclofenac sodium released from the formulations and are correlated with the disintegration time and the contact angle: increasing the concentration of the API increased the hydrophilicity of the films, allowing for a better wetting of the film with the dissolution medium (decrease in the contact angle) and rapid disintegration (decrease in the disintegration time), and ensuring the increased release of the diclofenac sodium.

The ability of maltodextrins to improve the dissolution rates of the active ingredients included in ODFs has been previously demonstrated [41]. Regarding the influence of the API on dissolution, a study in which the characteristics of diclofenac sodium-loaded, maltodextrin-based films were evaluated reported that the presence of diclofenac did not affect the dissolution rate of the API [23]. However, the study did not pursue variation in the diclofenac sodium concentration, and the information provided suggests that this API does not hinder the disintegration of the films and the subsequent dissolution.

Regarding the ODF preparation method, the authors of [25] found that the dissolution rate of piroxicam was clearly higher in the case of films prepared by solvent casting compared to films prepared by the HME method. However, it seems that not only the method led to these results but also the excipient in the formulation, namely, microcrystalline cellulose, which induced a slower disintegration of the films and, subsequently, a decrease in the piroxicam dissolution rate by two different mechanisms, swelling and delaying maltodextrin dissolution, thereby reducing its solubilizing effect [25]. The results obtained in our study are very promising, the API’s dissolution rate being comparable not only to those observed for films obtained by solvent casting but also to those observed in other experiments that studied ODFs prepared by HME [34,40,41,43].

### 2.6. Solid-State Analysis

#### 2.6.1. DSC Studies

As described in the Materials and Methods Section, the VCM process involves sample melting and thus thermal stress is applied. Thermal analysis was of paramount importance, first to select the melting temperature used during the VCM, and further to understand the changes that occur during the preparation process.

**Figure 5:**
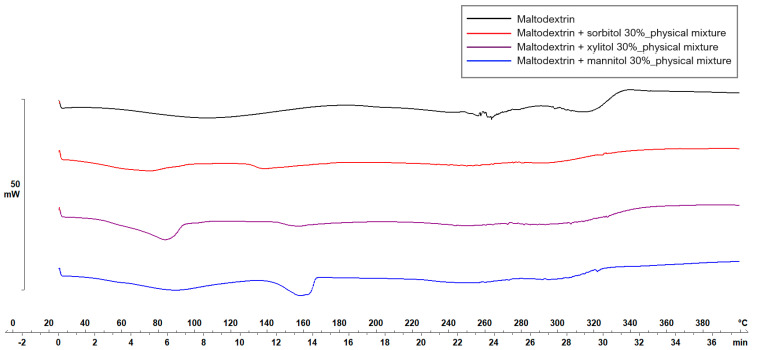
Thermal behavior evaluation for plasticizer selection.

**Figure 6:**
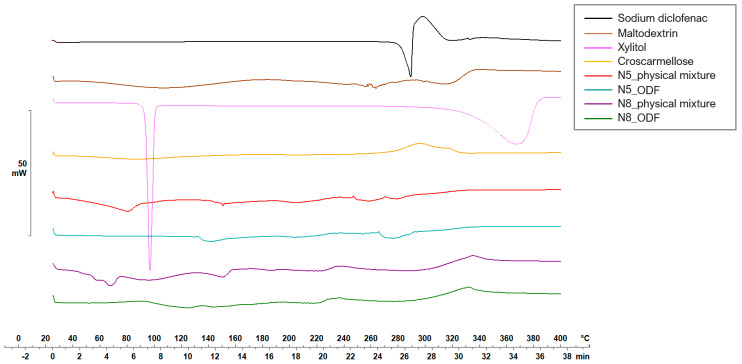
Thermal behaviors of the individual components of the ODFs and of the N5 and N8 physical mixtures and ODFs.

Figure 5 and Figure 6 display the thermograms corresponding to the pure ODF components, followed by the physical mixtures and the VCM ODF samples.

The MD displayed a large endothermic event between 38.7 and 178.06 °C, with the peak at 106.63 °C, which could be attributed to dehydration, although other authors have found the glass transition of MD at about the same temperature: 102.6 ± 2 °C [44]. Another endothermic peak was detected at 242.45 °C that could be associated with the melting phenomenon. Regarding the plasticizers, xylitol presented a sharp endothermic peak at about 94.42 °C, attributed to its melting [45,46]. The high melting enthalpy (1639.63 mJ) of xylitol is related to the cooling sensation it offers when dissolved in the oral cavity and is present only if xylitol maintains its crystalline form [45]. Sorbitol presented the endothermic peak at 100.71 °C, and mannitol at 166.04 °C (data not included). When MD was mixed with the plasticizers, namely, with xylitol, the endothermic peak that indicated the melting of the mixture appeared at about 160 °C (Figure 5). Therefore, the working conditions for the VCM ODF preparation were set at a temperature of 170 °C.

Diclofenac sodium presented a sharp endothermic peak at 298.59 °C, corresponding to its crystalline melting, followed by an exothermal phenomenon associated with the decomposition, all in agreement with other authors’ findings [47,48].

The physical mixtures displayed an initial narrow peak at 91 °C, corresponding to the melting of xylitol, followed by a broader peak at 152.65 °C, attributed to crystalline diclofenac sodium melting, and then a succession of endo-/exothermal phenomena associated mainly with decomposition. For both crystalline substances (i.e., xylitol and diclofenac sodium), a melting shift to a lower temperature was noticed, which can be attributed to the dilution effect or the partial dissolution of diclofenac sodium into MD. The VCM ODF thermograms displayed no crystalline peaks but rather a succession of broad endothermal and exothermal events with decomposition after about 180 °C, which could suggest the partially amorphous state of the components, including the initially crystalline xylitol and diclofenac sodium. 

#### 2.6.2. XRD Studies

**Figure 7:**
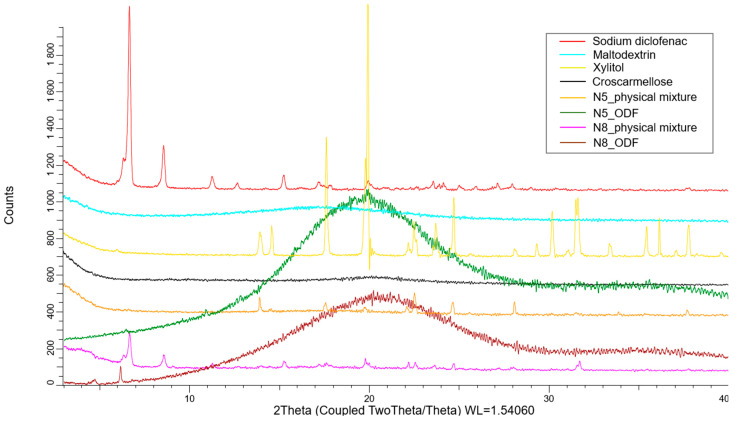
The X-ray diffractograms of the individual components of the ODFs and of the N5 and N8 physical mixtures and ODFs.

XRD was used to assess the crystalline state of the API in the physical mixtures, as well as in the VCM ODFs. As Figure 7 shows, the pure diclofenac sodium displayed many distinct peaks, among which those with the highest intensities were at 6.63°, 8.5°, and 15.2°. The amorphous maltodextrin and croscarmellose showed no distinct peaks. The physical mixtures containing all the components of the ODFs preserved the characteristic peaks of the API, but at a lower intensity due to the dilution phenomenon. Furthermore, the peaks corresponding to xylitol were visible. The diffractograms corresponding to the VCM ODFs presented no visible peaks, usually indicating amorphous contents and sustaining the previously discussed DSC evidence. Interestingly, the same appearance of the diffractogram was obtained for all the API doses, which showed that, even at high diclofenac sodium loading, MD can dissolve and turn it amorphous.

#### 2.6.3. FT-IR Studies

**Figure 8:**
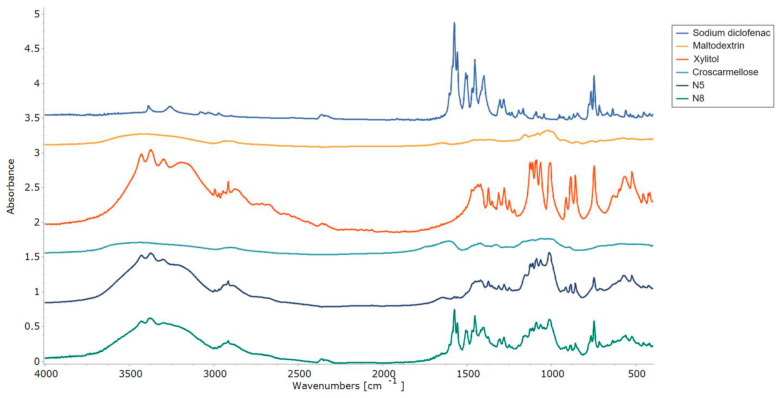
FT-IR patterns of the individual components of the ODFs and of the N5 and N8 ODFs.

The interactions between the ODF components were assessed by FT-IR experiments, and the resulting spectra are displayed in Figure 8. The pure diclofenac sodium showed bands representative of the symmetrical and asymmetrical stretching vibrations of the carboxylate groups at 1454 and 1580 cm^−1^. At 747 cm^−1^, the C-Cl stretching peak was visible [48], while at 3375 cm^−1^, a vibration band appeared for the secondary amino group [47]. FT-IR spectra showed the specific maltodextrin pattern with the two peaks at 992 cm^−1^ and 1012 cm^−1^, corresponding to the C-O stretching vibration. Meanwhile, for xylitol, its spectrum showed high-intensity vibrations at 3368 cm^−1^ and 3168 cm^−1^, which were attributed to O-H bonds [45]. Characteristic peaks corresponding to diclofenac sodium and xylitol were preserved in both VCM ODF spectra and were more evident in sample N8, due to the higher API loading.

#### 2.6.4. SEM Studies

**Figure 9:**
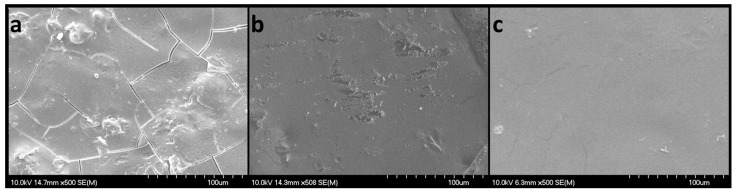
SEM micrographs of VCM ODFs: N4 (**a**), N5 (**b**), and N8 (**c**), captured with a magnification of 500×; scales represent 100 µm.

The SEM images (Figure 9) revealed flat, smooth, non-porous surfaces, with slight irregularities. When the scanning electron micrographs were examined for samples N4 (without diclofenac sodium), N5 (with 2.5 mg diclofenac sodium), and N8 (with 50 mg diclofenac sodium), their aspects were rather different. Sample N4 displayed small fractures that were not visible to the naked eye and could have appeared during manipulation. However, the observed fractures did not seem to harm the mechanical properties, as formulation N4 showed the highest peak loads in the puncture test. Sample N5 showed smooth surfaces with partial erosions that were also attributed to the manipulation and extraction from the VCM tool. Although sample N8 had the highest content of diclofenac sodium, no sign of recrystallization was noticed, only smooth plain surfaces.

#### 2.6.5. Raman Microscopy

**Figure 10:**
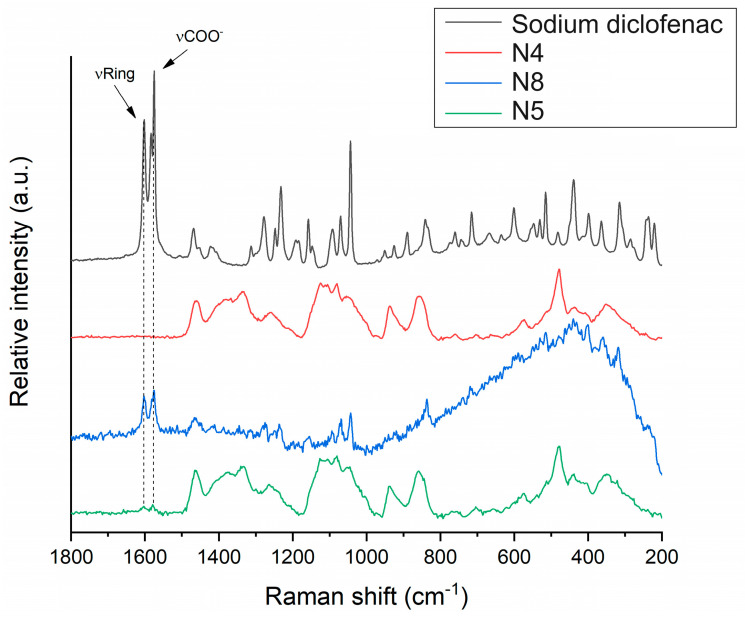
Raman spectra of diclofenac sodium in comparison to investigated ODF samples: diclofenac-free N4 reference, as well as diclofenac-loaded N8 and N5.

**Figure 11:**
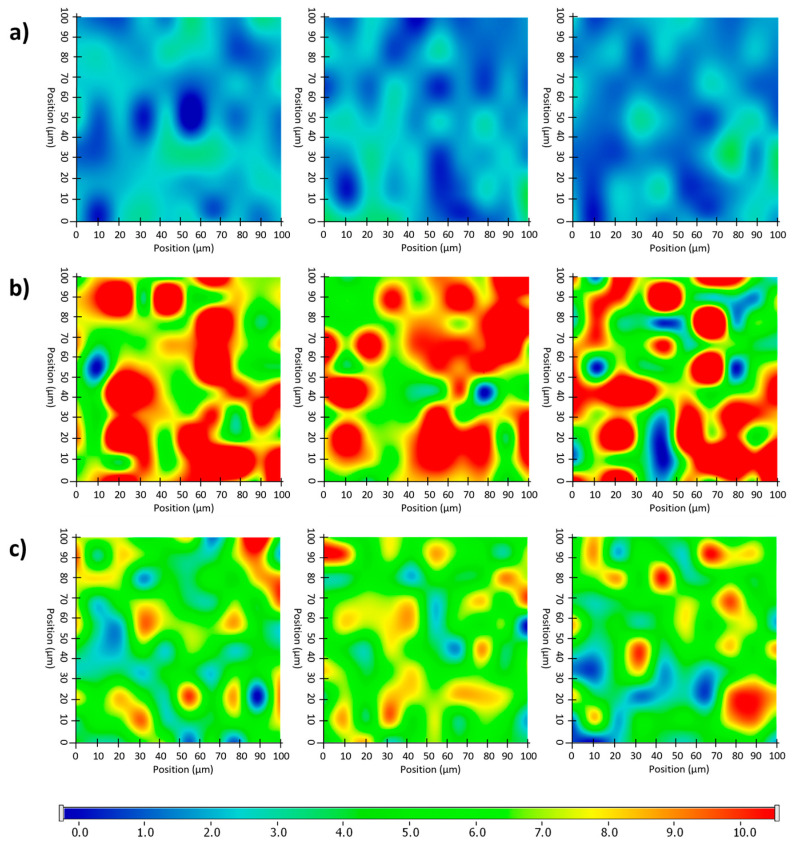
Randomly recorded Raman chemical maps of various ODF samples indicating the distribution and relative occurrence of diclofenac sodium: diclofenac sodium-free N4 reference (**a**), as well as diclofenac sodium-loaded N8 (**b**) and N5 (**c**).

The distribution of diclofenac sodium in the VCM ODF samples was determined using Raman mapping. For the investigation, the chemical structures of the pure drug and different ODF formulations were compared to find a unique spectral region in the Raman spectra, characteristic of the drug, to be further used as a reference for its localization in the ODF samples. For the diclofenac sodium profiling, the Raman peak at 1575 cm^−1^ assigned to the COO^-^ asymmetric stretching vibration and the band at 1603 cm^−1^ attributed to the dichlorophenyl and phenylacetate ring stretching vibrations were applied, according to [49] (Figure 10). For each ODF sample, three randomly selected surfaces with an area of 100 × 100 µm were examined (Figure 11).

After the structural examination of the initial components, Raman mapping was performed to investigate the distribution of the diclofenac sodium in the ODF samples. For the localization, the previously mentioned spectral regions were used as a reference, the frequencies of occurrence of which are shown on the chemical maps, representing the statistical distribution of the drug (Figure 11). The different colors of the chemical map indicate the relative intensity changes of the diclofenac sodium in the investigated ODFs. The red-colored areas indicate its strong presence, the green areas show a mixture of the API with other components, and the blue color marks those regions of the map with spectral resolutions containing different spectra, characteristic of another excipient. As a reference, a diclofenac sodium-free sample (N4) was investigated, as shown in Figure 11a. In this case, no specific drug characteristics were found, indicated by the blue and slightly green colors of the chemical map. For the diclofenac sodium-containing ODFs (N8 and N5), the distribution of diclofenac was homogenous, as shown by the well-defined patches of the yellow-red color (Figure 11b,c). Moreover, the intensity of the diclofenac sodium markers was remarkably higher in the case of sample N8 in comparison to that of N5, corresponding to the higher drug content in the former.

### 2.7. Practical Implications and Future Applications

VCM was initially applied in 2014 as a new technology designed for sample preparation to ease the development burden of ASDs. In more recent studies, it has been applied in different pharmaceutical products, particularly for solid dosage forms.

In the formulation of ODFs, VCM can be potentially employed individually or in conjunction with other techniques to address specific formulation challenges. During preformulation studies for ODF development, VCM can assist in the selection of excipients as a screening tool. As a small-scale preparation method, VCM is convenient due to its solvent-free nature, eliminating concerns about residual solvents and providing a faster alternative to solvent-casting methods. Additionally, its cost-effectiveness in terms of equipment costs compared to HME and the small amounts of APIs and excipients needed with reduced material loss make it affordable for small production units, like pharmacies and low-income areas. As shown in the current study, this method is able to deliver personalized dosage forms tailored to individual patient needs, from a minimum of three excipients. However, the main limitations of the ODF preparation process using VCM include its restriction to thermally stable APIs and raw materials, and its suitability only for small-batch production. Future research should focus on testing various APIs and excipients, improving the working procedure to enhance the reproducibility in the weight yield, as well as on increasing the productivity.

## 3. Materials and Methods

### 3.1. Materials

The chemicals used for the preparation of the ODFs were as follows: diclofenac sodium (pharmaceutical-grade), kindly donated by Aarti Drugs Ltd. (Mumbai, India); maltodextrin with a dextrose equivalent of a maximum of 19% (Glucidex^®^ Premium IT 19, Roquette, Lestrem, France) as a film-forming agent; sorbitol (D-Sorbitol ≥ 98%, Sigma-Aldrich, St Louis, MO, USA), xylitol (Xylisorb^®^ 300, Roquette, Lestrem, France), and mannitol (Parteck M200, Merck, Germany) as plasticizers; and croscarmellose (Dislocel^®^, Mingtai Chemical Co, Ltd., Taoyuan, Taiwan) as a superdisintegrant. Commercial ODFs were purchased from community pharmacies: Melatonin Pura Fast (ESI s.p.a., Euronet Growth Milan, Italy) (Commercial Film 1, CF1) and Vitamin D3 2000 NE (IBSA Farmaceutici, Lodi, Italy) (Commercial Film 2, CF2). All other chemicals were analytical-grade.

### 3.2. Sample Preparation

**Figure 12:**
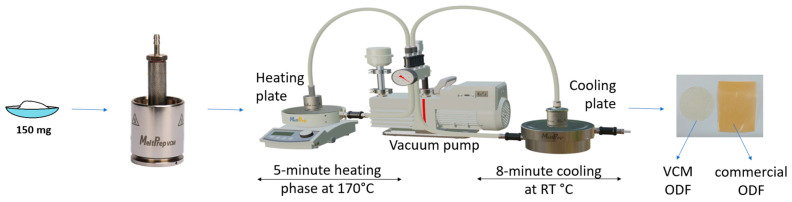
Graphical representation of the VCM ODF preparation steps.

An amount of 10 g of each powder blend, with the compositions shown in Table 4, were prepared by manually grinding and mixing the materials for 3 min in a mortar. For the VCM ODF preparation, 150 mg of mixture was loaded into the circular chamber (VCM disc tool with 20 mm diameter) of the Vacuum Compression Molding equipment (MeltPrep, Graz, Austria). Vacuum was applied, and then the insert was placed onto the heating plate for 5 min and kept at 170 °C. Afterwards, the sample was moved onto the cooling plate coupled with a source of compressed air for 8 min at room temperature. The schematic representation of the ODF preparation process is given in Figure 12. The resulting ODFs were then extracted and kept in the desiccator until further use. A total of 20 samples were prepared for each formulation. The medium weight of each ODF sample was 150 mg. The diclofenac sodium doses for samples N5–N8 were 2.5 mg, 5 mg, 10 mg, and 50 mg, respectively.

### 3.3. Mechanical Characterization

Two tests were applied for the mechanical characterization of the films using a CT3 texture analyzer (Brookfield Ametek, Middleboro, MA 02346, USA), equipped with a 4.5 kg load cell.

Firstly, a puncture test was applied using a film fixture with a 10 mm diameter orifice (TA-RT-KIT) and a cylinder rig (2 mm diameter) (TA3/100) that descended through the sample at a speed of 0.10 mm/s after a trigger load of 5 g was attained.

Secondly, a tensile test was performed using a dual-grip rig (TA-DGF) that ascended at a speed of 0.10 mm/s and a trigger load of 1 g up to a target distance of 3 mm. The thickness was measured before the texture analysis using a digital caliper for each sample. All tests were performed in triplicate, and the load-versus-distance profiles were plotted. Texture Pro CT software (Brookfield Ametek, Middleboro, MA, USA) was used to calculate the following mechanical parameters: the load at puncture/at target, deformation at puncture/at target, and fracturability. Furthermore, the puncture strength and breaking factor were calculated from the profiles revealed by the puncture tests, according to literature-reported equations [28,29]. The tensile test enabled the calculation of the tensile strength, elongation at peak load, and Young’s Modulus [28].

### 3.4. Contact Angle Measurement

The water contact angle was used as an instrument to evaluate the wettability of the ODF samples. The measurements were carried out with the drop-shape analyzer OCA 25 (Dataphysics Instruments, Stuttgart, Germany), using distilled water drops of 2.5 µL. Three drops were placed on each film in different places, and the software of the drop analyzer automatically calculated the angle for each measurement. The contact angle was measured at 0.05 ± 0.01 s after the drop landing.

### 3.5. Disintegration Testing

Many research groups use the pharmacopeial disintegration test to determine whether ODFs disintegrate within the prescribed time: 3 min [41]. Therefore, the disintegration test of the ODFs was carried out according to the specifications of the monograph “2.9.1. Disintegration of tablets and capsules” reported in the Ph. Eur., using the Erweka ZT 120 disintegration tester (Erweka GmbH, Langen, Germany). Randomly selected films of each formulation were analyzed in distilled water maintained at 37 ± 0.5 °C. The disintegration time was recorded when no residue remained in the mesh of the basket-rack assembly.

**Figure 13:**
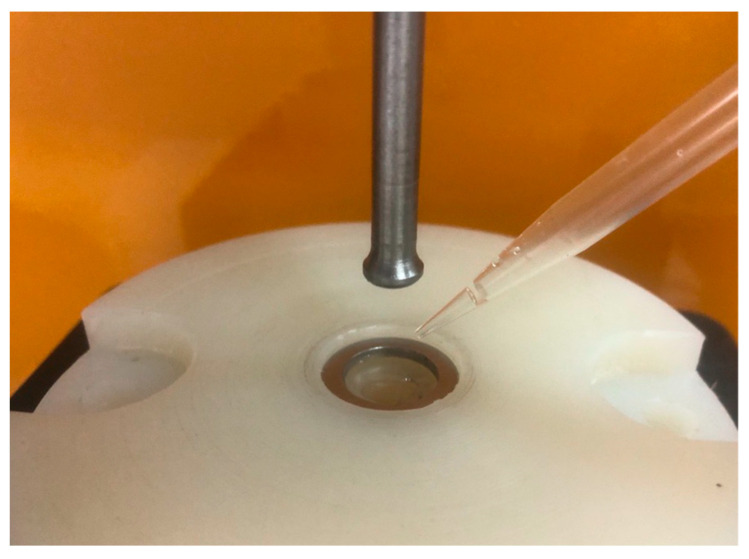
Experimental setup of the disintegration test performed by texture analysis.

To simulate the oral conditions (e.g., a low saliva volume available for disintegration, mechanical stress applied by the tongue), the disintegration time was also evaluated through texture analysis, as shown in Figure 13. The method was adapted after [50] and involved the use of a puncture test fixture with a 10 mm orifice [50]. The VCM ODFs were placed in the sample holder and fixed with a 20 mm diameter ring. Each sample was wetted with 200 µL artificial saliva, and after a lag time of 5 s, an 8 mm round-ended cylindrical probe descended through the sample at a speed of 0.1 mm/s, down to a target distance of 10 mm. The load-versus-time profiles were recorded, and the disintegration times were calculated as the difference between the time when the probe reached the minimal sample resistance (disintegration endpoint) and the time of the peak load (marking the beginning of the disintegration) [50]. The disintegration test for both methods was performed in triplicate for each formulation, and the results were expressed as mean ± S.D.

### 3.6. Dissolution Studies

The dissolution studies were performed according to the Ph. Eur. 11.5 Ed. using a paddle dissolution apparatus in 900 mL phosphate buffer, pH 6.8, at 37 °C and a 50 rpm rotation speed, on accurately weighed ODFs. We withdrew 5 mL samples at 5, 10, 15, 20, 30, and 60 min throughout the test and replaced them with 5 mL of fresh medium. Diclofenac sodium was assayed through a validated HPLC method on an Agilent 1100 Series HPLC system (Agilent Technologies, Santa Clara, CA, USA), using an UV detector at 278 nm and a Luna C18(2) column (5 μm, 150 × 4.6 mm, 100 Å), a mobile phase composed of phosphoric acid 0.1% and acetonitrile 25:75 (*v*/*v*), and a flow of 1 mL/min, at a retention time of 2.9 min. The dissolution test was performed in triplicate for each formulation.

### 3.7. Solid-State Analysis

#### 3.7.1. Differential Scanning Calorimetry (DSC)

DSC analysis was performed with a DSC3 Star^e^ System (Mettler Toledo GmbH, Zurich City, Switzerland). Samples of 2–3 mg of the pure substances, their physical mixtures, and of the VCM ODFs were accurately weighed into 40 µL aluminum pans and sealed with pierced lids. Further, they were analyzed under a dynamic nitrogen atmosphere (50 mL/min) over a temperature range of 25–400 °C with a heating rate of 10 °C/min. The resulting thermograms were analyzed using STAR SW 12.10 software (Mettler Toledo GmbH, Switzerland).

#### 3.7.2. X-ray Diffraction (XRD)

XRD analysis was performed to investigate the physical state of the diclofenac sodium in the different stages of the preparation process: on the pure substance, on the physical mixtures, and on the VCM ODFs. XRD spectra were recorded with a BRUKER D8 Advance X-ray diffractometer (Bruker AXS GmbH, Karlsruhe, Germany) system with Cu Kα1 radiation (λ = 1.5406 Å) over the range 5–40°/2°. The following experimental setup was employed: target: Cu; filter: Ni; voltage: 40 kV; current: 40 mA; time constant: 0.1 s; angular step: 0.010°.

#### 3.7.3. Scanning Electron Microscopy (SEM)

The morphologies of the films were evaluated through SEM (Hitachi S4700, Hitachi Scientific Ltd., Tokyo, Japan) by applying a potential of 10 kV and 1.3–13.0 mPa air pressure. Before the analysis, samples were sputter-coated with gold–palladium to ensure conductivity (Bio-Rad SC 502, VG Microtech, Uckfield, UK).

#### 3.7.4. Raman Microscopy

Raman mapping of the ODF samples was performed using a Thermo Fisher DXR dispersive Raman instrument (Thermo Fisher Scientific Inc., Waltham, MA, USA) equipped with a CCD camera and a diode laser operating at a wavelength of 780 nm. For the analysis, ODF samples were mounted on a glass slide that was covered with aluminum foil. Raman chemical maps were recorded from 100 × 100 µm surfaces of different ODFs with a 10 µm step size, while applying a laser power of 24 mW at a 50 µm slit aperture size. The spectrum of the chemical map was recorded with an exposure time of 2 s and an acquisition time of 6 s, for a total of 16 scans per spectrum in the spectral range 3300–200 cm^−1^, with cosmic ray and fluorescence corrections. Each Raman map was normalized in order to eliminate the intensity deviation between the measured areas.

## 4. Conclusions

This study was a proof-of-concept study to demonstrate the feasibility of using VCM as an ODF preparation method. This technology enables the preparation of thermoplastic dosage forms with personalized dosing, a feature that is especially useful for pediatric patients but that is not limited only to them.

Briefly, diclofenac sodium-loaded ODFs for pediatric use were successfully formulated and prepared by the VCM technique, using maltodextrin as the film-forming polymer. The study’s objectives were to perform an initial selection of the plasticizer, to add an appropriate amount of disintegrant, and, finally, to add different concentrations of the API (2.5 mg, 5 mg, 10 mg, and 50 mg of diclofenac sodium) in order to evaluate the influence of each formulation factor on the quality of the ODFs, and to demonstrate that VCM is suitable for personalized medicine preparation. The mechanical characterization of the ODFs revealed that xylitol was the best plasticizer, contributing to the formation of flexible ODFs. Regarding the disintegrant, the mechanical tests showed a further improvement in terms of the flexibility and resistance to fractures. While it is true that adding the API resulted in less ductile films, their mechanical properties are comparable to those of authorized commercial products and the data in the literature. All the characterization methods applied demonstrated the obtention of high-quality, wettable films with smooth surfaces, good distributions of the API in the ODFs, good disintegration capacities, and the release of more than 85% of the API within 5 min, regardless of the API loading dose. Therefore, VCM shows great potential in functioning as a method of preparing small-scale, personalized dosage forms.

Some of the advantages are presented in Section 2.7; however it must be added that this method could find application both in community and hospital pharmacies as a cost-effective technique, providing flexibility in dosing based on the individual patient characteristics and medical conditions. Additionally, tailoring doses to individual needs has the potential to reduce the risk of adverse effects.

## Figures and Tables

**Table 1:** Physical characteristics of the ODFs.

	Appearance	Thickness (µm)	Weight Yield (%)		Appearance	Thickness (µm)	Weight Yield (%)
**N1**	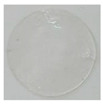	320 ± 46.4	85.12 ± 9.07	**N5**	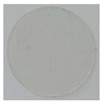	313.3 ± 21.6	94.83 ± 2.53
**N2**	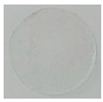	241.6 ± 27.8	66.33 ± 12.38	**N6**	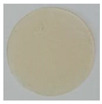	316.6 ± 10.3	92.65 ± 5.34
**N3**	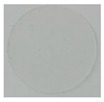	270 ± 27.3	79.87 ± 9.52	**N7**	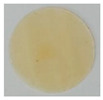	306.6 ± 19.6	92.24 ± 5.94
**N4**	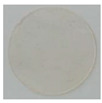	313.3 ± 26.4	94.22 ± 5.82	**N8**	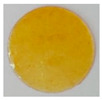	308.3 ± 19.6	88.66 ± 7.47

**Table 2:** Mechanical characterization results for the VCM ODFs.

	Puncture Test					Tensile Test		
	Load at Puncture (g)	Deformation at Puncture (mm)	Fracturability (g)	Puncture Strength (N/mm^2^)	Breaking Factor (N/mm)	Tensile Strength (MPa)	Elongation at Peak Load (%)	Young’s Modulus (MPa)
**N1**	300.30 ± 109.80	0.88 ± 0.37	97.50 ± 20.10	0.92 ± 0.35	9.2 ± 3.49	1.80 ± 0.49	111.33 ± 7.29	41.34 ± 1.04
**N2**	94.50 ± 9.00	0.33 ± 0.25	70.80 ± 26.00	0.37 ± 0.08	3.73 ± 0.76	2.76 ± 0.87	109.17 ± 2.25	45.33 ± 1.23
**N3**	160.50 ± 19.50	0.09 ± 0.04	149.20 ± 35.60	0.57 ± 0.06	5.71 ± 0.64	0.78 ± 0.29	103.5 ± 0.71	30.85 ± 12.45
**N4**	397.50 ± 125.30	0.80 ± 0.12	299.30 ± 196.50	1.17 ± 0.39	11.68 ± 3.91	1.86 ± 0.56	134.33 ± 7.97	29.30 ± 6.87
**N5**	261.50 ± 140.4	0.96 ± 0.37	218.30 ± 177.70	0.58 ± 0.10	5.77 ± 0.97	2.03 ± 0.59	135.5 ± 11.95	37.62 ± 5.61
**N6**	180.50 ± 5.66	0.38 ± 0.26	146.5 ± 11.80	0.49 ± 0.01	4.87 ± 0.12	2.86 ± 0.52	119.17 ± 5.97	46.14 ± 5.61
**N7**	145.25 ± 1.77	1.04 ± 0.42	114.50 ± 39.50	0.46 ± 0.006	4.59 ± 0.06	2.96 ± 0.12	110.83 ± 3.06	52.83 ± 11.10
**N8**	243.80 ± 65.80	0.83 ± 0.18	142.80 ± 68.70	0.78 ± 0.14	7.82 ± 1.39	2.32 ± 0.34	109.33 ± 0.29	49.92 ± 5.48

**Table 3:** Wettability and disintegration behavior of ODFs.

	Contact Angle (°)	Disintegration Time— Ph. Eur. Method (s)	Disintegration Time— Texture Analysis Method (s)
N1	52.14 ± 2.38	60.00 ± 0	51.42 ± 8.50
N2	47.39 ± 9.77	60.00 ± 0	50.27 ± 11.24
N3	42.10 ± 4.72	70.00 ± 0	62.13 ± 10.92
N4	47.44 ± 6.08	68.33 ± 7.64	59.57 ± 12.50
N5	64.83 ± 7.98	101.33 ± 10.26	65.33 ± 10.27
N6	58.43 ± 7.70	101.67 ± 20.21	57.93 ± 18.01
N7	48.89 ± 6.82	68.00 ± 5.20	46.53 ± 8.38
N8	47.90 ± 5.49	83.67 ± 10.12	41.00 ± 14.25

Mean ± S.D. (n = 3).

**Table 4:** ODF composition.

	N1	N2	N3	N4	N5	N6	N7	N8
Maltodextrin (%)	70	70	70	65	63.34	61.67	58.34	31.67
Sorbitol (%)	30							
Xylitol (%)		30		30	30	30	30	30
Mannitol (%)			30					
Croscarmellose sodium (%)				5	5	5	5	5
Diclofenac sodium (%)					1.66	3.33	6.66	33.33

## Data Availability

The raw data supporting the conclusions of this article will be made available by the authors upon request.
